# The role of musculoskeletal radiologists in emergency and trauma settings: current and emerging imaging modalities

**DOI:** 10.1093/bjro/tzaf025

**Published:** 2025-11-02

**Authors:** Muhammad Israr Ahmad, Lulu Liu, Adnan Sheikh, Savvas Nicolaou

**Affiliations:** Department of Radiology, Faculty of Medicine, University of British Columbia, Vancouver, BC V5Z 1M9, Canada; Department of Radiology, Faculty of Medicine, University of British Columbia, Vancouver, BC V5Z 1M9, Canada; Department of Radiology, Faculty of Medicine, University of British Columbia, Vancouver, BC V5Z 1M9, Canada; Department of Radiology, Faculty of Medicine, University of British Columbia, Vancouver, BC V5Z 1M9, Canada

**Keywords:** ER & Trauma Radiology, MSK Trauma Imaging

## Abstract

MSK radiologists play a critical role in emergency and trauma settings, where rapid and accurate imaging interpretation is essential for timely diagnosis and treatment. The increasing complexity of trauma cases has driven the adoption of advanced imaging modalities beyond conventional radiographs and computed tomography (CT). Dual-energy CT (DECT) and magnetic resonance imaging (MRI) have revolutionized MSK imaging, offering superior tissue characterization and improved detection of occult fractures, bone marrow edema (BME), infections, and soft tissue injuries. Emerging technologies, such as portable MRI and photon-counting CT (PCCT), further enhance diagnostic capabilities by enabling bedside imaging, reducing radiation exposure, and providing ultra-high-resolution images. MSK radiologists are integral to immediate diagnosis, triaging, differentiating acute from chronic injuries, guiding surgical interventions, and performing image-guided procedures. DECT in particular has proven invaluable in detecting BME, reducing metal artifacts, and improving soft tissue contrast, while MRI remains the gold standard for evaluating soft tissue injuries and occult fractures. Portable MRI offers a radiation-free alternative for point-of-care imaging, especially in spinal cord and soft tissue injuries. PCCT, with its superior spatial resolution and material decomposition capabilities, holds promise for advanced fracture detection and reduced radiation doses. Additionally, 3D printing has emerged as a transformative tool for preoperative planning, surgical simulation, and personalized implant design. Despite challenges such as cost, accessibility, and technical limitations, these advancements are reshaping trauma imaging. As technology evolves, MSK radiologists will continue to integrate these innovations to optimize patient care in emergency and trauma settings, ensuring faster, more accurate diagnoses.

## Introduction

Musculoskeletal (MSK) radiologists are essential members of the emergency and trauma care team, where quick and accurate interpretation of imaging can make a critical difference in patient outcomes. Trauma cases are often complex, with patients presenting multiple injuries that require fast, informed decisions. To meet these demands, radiologists increasingly rely on advanced imaging techniques that go beyond standard X-rays and conventional CT scans, allowing them to identify subtle injuries that might otherwise be missed and directly guide patient management.^1–3^

Dual-energy CT (DECT) and MRI have greatly expanded the capabilities of MSK radiologists. DECT has improved tissue characterization, making it easier to detect subtle fractures, bone marrow edema (BME), and soft tissue injuries. It is also effective at reducing metal artifacts, which is especially useful in patients with implants or prior orthopedic surgery. MRI continues to be the gold standard for assessing ligaments, tendons, occult fractures, bone marrow changes, and infections. Its high contrast resolution and ability to image in multiple planes make it invaluable for evaluating complex MSK trauma that may not be fully appreciated on CT or X-rays.^2^^,^^4^

Portable MRI is an exciting development, allowing imaging at the bedside without the need for conventional high-field MRI scanners. Using low-field magnets, portable MRI provides rapid imaging for critically ill or immobile trauma patients in emergency departments (EDs), ICUs, or remote locations. This makes it easier to identify fractures, soft tissue injuries, and joint abnormalities while minimizing logistical challenges and delays.^5^

Photon-counting CT (PCCT) is another emerging technology that promises even higher-resolution imaging. PCCT produces images with improved contrast and lower noise, while also reducing radiation exposure. It is particularly helpful for evaluating fine bone structures, subtle fractures, and post-surgical anatomy, offering a level of detail previously unattainable with conventional CT.^3^^,^^6^

Together, these advanced imaging tools empower MSK radiologists to triage patients effectively, differentiate acute from chronic injuries, guide surgical planning, and perform image-guided procedures. By incorporating DECT, MRI, portable MRI, and PCCT into clinical practice, trauma teams can achieve faster, more accurate diagnoses and make better-informed treatment decisions, ultimately improving patient outcomes.

## The role of MSK radiologists in the ER and trauma settings

### Immediate and accurate diagnosis

One of the primary responsibilities of MSK radiologists in the ED is the prompt and accurate interpretation of imaging studies. Trauma patients often present with complex injuries that require detailed assessment, including fractures, ligamentous injuries, dislocations, and soft tissue damage. Timely identification of these conditions ensures appropriate management, whether surgical or conservative.

In particular, occult fractures, such as femoral neck fractures, scaphoid fractures, and tibial plateau fractures, can be missed on initial radiographs. MSK radiologists utilize advanced imaging modalities, such as DECT and MRI, to detect these injuries, preventing delayed diagnoses that could lead to poor clinical outcomes.^7–9^

### Triaging and prioritizing cases

Emergency settings often involve multiple trauma patients with varying degrees of severity. MSK radiologists play a key role in triaging injuries, prioritizing patients who require immediate surgical intervention while identifying those who can be managed conservatively.

For instance, in polytrauma cases, rapid whole-body CT (WBCT) scans are performed to assess injuries to the head, chest, abdomen, and extremities. MSK radiologists must quickly differentiate between life-threatening fractures, such as unstable pelvic fractures, and those that are less urgent but still require orthopedic follow-up.^10^

### Differentiating acute from chronic injuries

MSK radiologists must distinguish between acute and chronic injuries, as misclassification can lead to unnecessary interventions. For example, in spinal trauma, DECT and MRI are used to differentiate acute vertebral compression fractures, which exhibit BME, from chronic fractures, which do not. This differentiation is crucial in deciding whether a patient requires immediate spinal stabilization or can be managed conservatively.^11^

### Guiding orthopedic and trauma surgeons

Accurate imaging interpretation assists orthopedic and trauma surgeons in making informed decisions regarding fracture fixation, ligament repair, and other surgical interventions. 3D reconstructions from CT scans allow better visualization of complex fractures, particularly in the acetabulum, scapula, and calcaneus, enabling more precise surgical planning.^12^

### Performing image-guided interventions

MSK radiologists are also involved in image-guided procedures,^13^ such as:

- Joint aspirations and drain placements for septic arthritis or abscesses.
- Minimally invasive fracture management, such as percutaneous vertebroplasty for osteoporotic fractures.
- Nerve blocks and pain management procedures in trauma patients with severe musculoskeletal pain.

## DECT in trauma and emergency settings

DECT is a cutting-edge imaging technology that enhances the detection and characterization of musculoskeletal injuries by utilizing two different X-ray energy levels (typically 80 and 140 kVp). This technique allows for material decomposition, improved contrast resolution, and reduction of artifacts, making it particularly useful in trauma and emergency settings. These images are then processed using specialized algorithms to extract additional information that would not be visible on standard CT scans. This allows for enhanced visualization of different tissue types, such as bone, cartilage, and soft tissues, which are critical in assessing various injuries in trauma patients.^1^^,^^2^

### BME detection: a game-changer in trauma imaging

BME is an important indicator of occult fractures, bone contusions, and early stress injuries. Traditionally, MRI has been the gold standard for detecting BME using fluid-sensitive sequences (STIR and fat-suppressed T2-weighted imaging). However, MRI is not always available in emergency settings, and not all patients can undergo an MRI (eg, those with pacemakers or severe claustrophobia).

**Figure 1.**
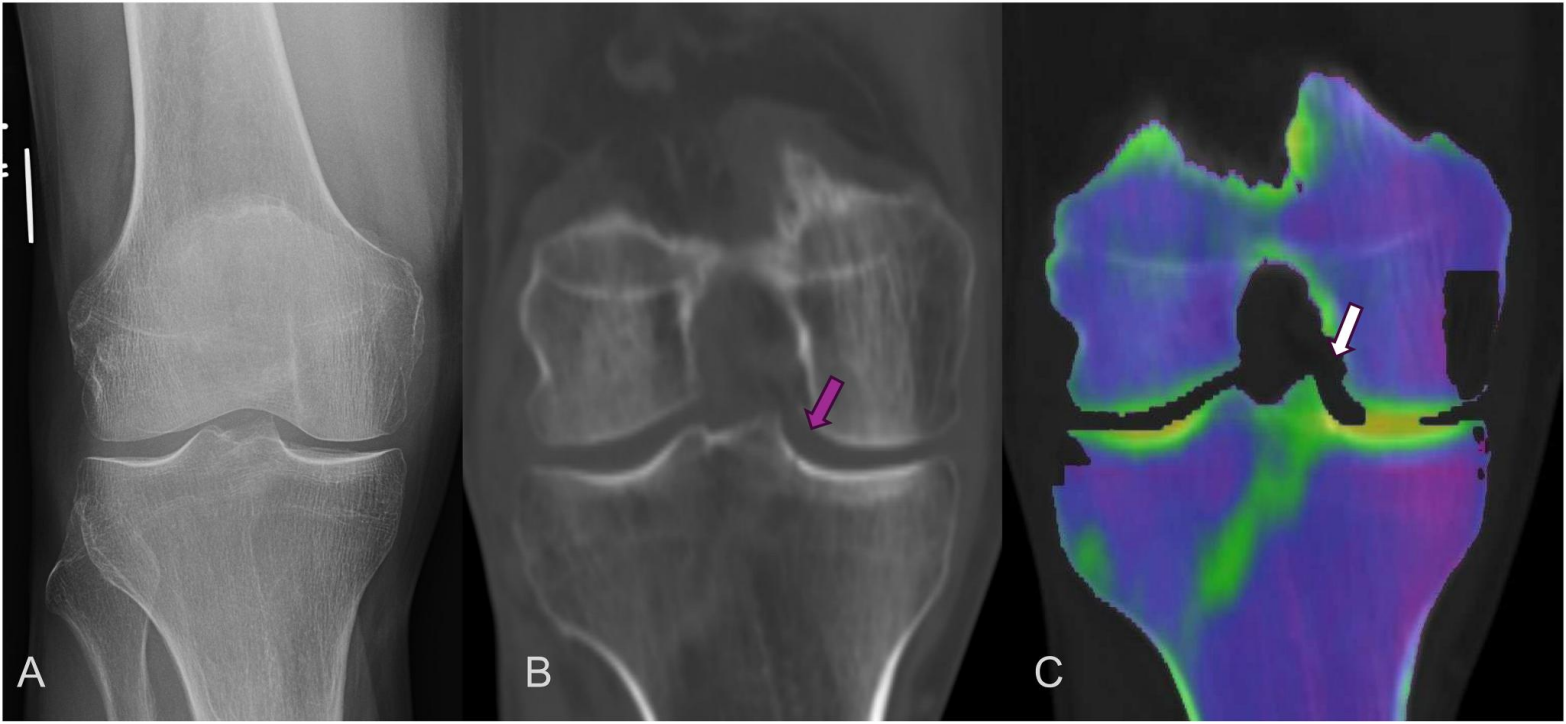
The radiograph of the patient presented in ER was negative for any acute fracture (A) however DECT was performed which showed an incomplete fracture on CT (B) but on dual energy map there was linear marrow edema involving the lateral tibial plateau (C).

DECT can generate virtual non-calcium images, which subtract calcium-containing structures (cortical and trabecular bone), highlighting areas of increased water content. These areas correspond to BME, making DECT a valuable alternative to MRI in acute trauma cases^1^^,^^2^ (Figure 1).

**Applications:** 

- *Spinal Trauma*: Differentiating acute vertebral fractures (BME present) from chronic fractures (no BME).
- *Pelvic and Hip Injuries*: Identifying occult femoral neck fractures, particularly in elderly patients with osteoporosis.
- *Extremity Trauma*: Detecting stress fractures in athletes, which might be invisible on conventional radiographs.

### Infection and inflammatory changes

**Figure 2.**
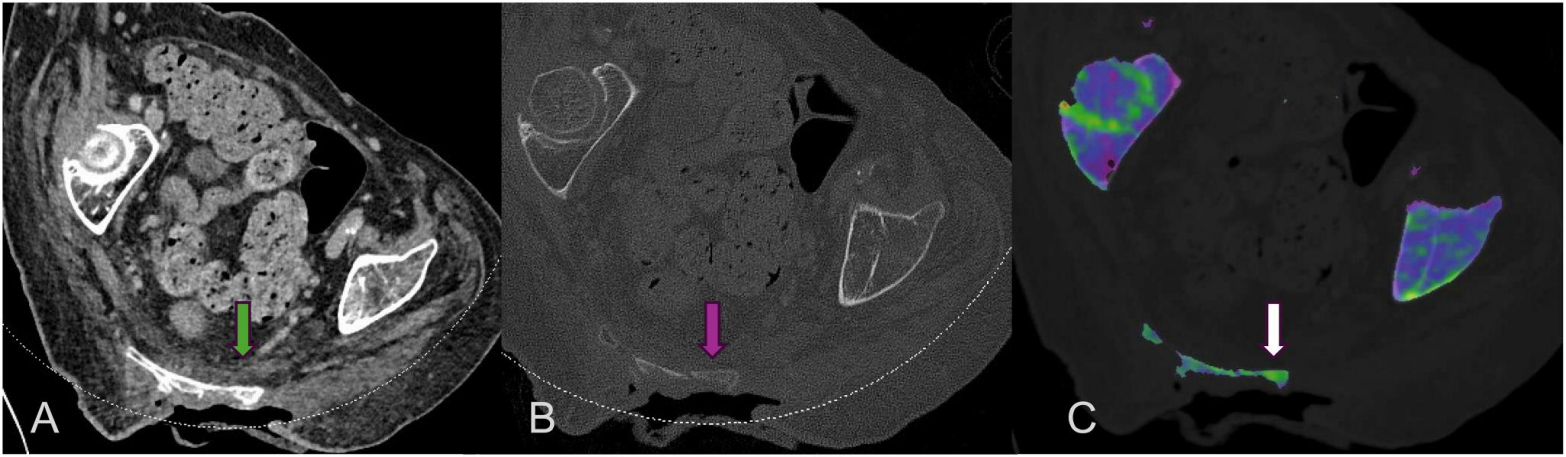
Dual energy CT of a patient was performed for clinical question of osteomyelitis. The patient had a sacral ulcer (Figure 2A & B). Dual energy maps confirmed the presence of marrow edema (Figure 2C) within the sacrum in keeping with osteomyelitis.

Septic arthritis, osteomyelitis, and soft tissue infections require early diagnosis and treatment to prevent devastating complications^14^^,^^15^ (Figure 2). DECT enhances infection detection by:

- Improving visualization of periosteal reactions and subperiosteal abscesses.
- Enhancing iodine mapping, which highlights hypervascular inflammatory changes.
- Reducing metal artifacts, allowing better assessment of peri-prosthetic infections in patients with joint replacements.

### Tendon, ligament, and meniscal injuries

While MRI remains the gold standard for evaluating soft tissue injuries, DECT provides a rapid alternative when MRI is unavailable. Tendons and ligaments, due to their relatively low intrinsic contrast on single-energy CT scans, can be difficult to assess for injury. DECT improves soft tissue contrast by differentiating between the water content, collagen, and fat in tendon and ligament tissues. This increased contrast allows for better visualization of subtle injuries, including tears, sprains, or inflammation, that might not be detectable on standard CT scans.^16^^,^^17^

Tendons, especially in chronic injury settings, may develop calcifications or ossifications. DECT can more clearly distinguish between different types of calcifications (such as those found in tendon calcifications, as seen in tendinopathies like calcific rotator cuff tendinopathy) compared to traditional CT. This enables more precise assessment of calcific deposits that may be a contributing factor to pain or dysfunction.^18^

DECT can also aid in distinguishing between different types of soft tissue lesions that may mimic tendon and ligament injuries, such as soft tissue tumors, cysts, or inflammatory conditions like bursitis or synovitis. By improving the contrast resolution of soft tissues, DECT offers an advantage in identifying these conditions with greater specificity, reducing the need for additional imaging modalities like MRI.

DECT is increasingly used in trauma and sports medicine for the assessment of tendon and ligament injuries. It can help identify tears, sprains, and strains in the setting of acute trauma or overuse injuries. The ability to detect accompanying bone injuries, such as fractures or bone contusions, in the same scan is particularly beneficial for comprehensive trauma evaluation.^15^

Following tendon or ligament repair surgeries, DECT can provide valuable information regarding the healing process. By assessing the soft tissue interface and the presence of any postoperative complications (such as infection, hematomas, or incomplete healing), DECT can help in monitoring recovery and identifying any issues that may require further intervention.

### DECT in nontraumatic emergencies

In nontraumatic emergencies, DECT is particularly useful in detecting gout by visualizing urate crystal deposition and distinguishing it from other causes of acute monoarthritis. MSK radiologists help differentiate true crystal arthropathy from degenerative or infectious processes, expediting appropriate treatment.^19^

### Metal artifact reduction

In MSK radiology, particularly in emergency and trauma settings, metal artifact reduction is a crucial challenge. Patients with orthopedic implants, such as joint replacements, fracture fixation devices, or prosthetics, often require imaging to assess injuries, infections, or complications. However, the presence of metal in the body can generate significant artifacts in conventional CT scans, which hinder the accurate interpretation of soft tissues and bony structures surrounding the metal. DECT has emerged as a promising tool to mitigate these metal artifacts, offering a significant advantage in trauma and MSK radiology.^20^^,^^21^

Metal artifacts in conventional CT occur due to the high atomic number of metal, which significantly attenuates the X-rays passing through it. This results in streaking, shadowing, and distortion of surrounding tissues, making it difficult to evaluate critical anatomical details, such as bone fractures, joint spaces, soft tissue injuries, or infections. This is particularly problematic in trauma settings where fast and accurate imaging is needed to assess fractures, infections, and injuries adjacent to metal implants.

One of the most significant advantages of DECT is its ability to perform material decomposition. This process allows DECT to separate materials based on their atomic composition, specifically distinguishing between metals and surrounding tissues. Through material decomposition, DECT can reduce the impact of metal by isolating and removing metal-related artifacts from the final image, leading to clearer visualization of soft tissues and bony structures.

DECT leverages the difference in X-ray attenuation at different energy levels to reduce artifacts. The energy spectrum of high-energy X-rays is more attenuated by metal, while low-energy X-rays are less affected. By combining the information from both energy levels, DECT can generate images that provide better contrast in areas surrounding metal implants, reducing the streaks and distortions typical in conventional CT scans.

DECT systems employ specialized software and algorithms designed to reduce artifacts through iterative reconstruction methods. These algorithms help minimize the streaking caused by metal by reconstructing images that better represent the surrounding anatomy, improving diagnostic accuracy in MSK radiology.^22^

Patients who have multiple orthopedic implants, such as those who have undergone joint replacements or complex trauma surgery, may benefit from DECT’s ability to reduce the cumulative metal artifacts caused by several implants. DECT helps in achieving better clarity and precision in these cases, enabling accurate assessments of both implants and the surrounding anatomical structures.^23^

For patients who require revision surgery, accurate preoperative planning is essential. DECT allows surgeons to better visualize the anatomy surrounding the metal implants, facilitating the planning of the most appropriate surgical approach. The clearer imaging provided by DECT can help avoid damage to critical structures and ensure more effective surgical interventions.

### Limitations of dual energy CT

DECT has shown considerable potential in identifying BME and subtle fractures, but it does have limitations, especially in the acute trauma setting. Its sensitivity can be reduced in anatomically complex regions, such as the spine and pelvis, where beam-hardening and metal artifacts may obscure small marrow changes.^24^ DECT may also struggles to distinguish acute from chronic marrow abnormalities, since signal alterations can persist beyond the acute phase and sometimes produce false-positive findings.^25^ Additionally, variations in post-processing algorithms and the experience of the reader can influence diagnostic accuracy, making reproducibility across institutions a challenge. Despite these limitations, DECT remains a valuable tool in acute trauma evaluation, particularly when MRI is unavailable or contraindicated, but its findings should always be interpreted in the context of clinical and other imaging data.

## WBCT in trauma setting

Whole body CT is now widely regarded as the gold standard for the initial assessment of polytrauma patients in the ED, as it allows for a rapid and comprehensive evaluation of intracranial, thoracoabdominal, and musculoskeletal injuries in a single scan. In this fast-paced setting, MSK radiologists play an essential role in triaging patients by providing accurate and timely interpretation of musculoskeletal findings that can directly impact clinical decision-making. While WBCT is often performed with the primary aim of identifying life-threatening injuries to the head, chest, or abdomen, research has shown that most traumatic injuries involve the musculoskeletal system, with fractures and joint disruptions contributing significantly to morbidity.^26^^,^^27^ MSK radiologists bring specialized expertise in detecting subtle fracture lines, complex pelvic or spinal injuries, and indirect signs of ligamentous or soft-tissue damage—findings that may be missed by non-specialists during the initial survey. Their input not only minimizes the risk of missed or delayed diagnoses but also supports the appropriate prioritization of surgical intervention, orthopedic referral, or conservative management. In addition, MSK radiologists play a key role in optimizing WBCT protocols to ensure adequate skeletal coverage while avoiding unnecessary radiation exposure, particularly in younger patients. They also contribute during multidisciplinary trauma team discussions by helping to stratify injuries according to their urgency and stability.^28^ In this way, within WBCT-based trauma assessment, MSK radiologists improve diagnostic accuracy, streamline decision-making, and ultimately enhance patient outcomes.

### Role of 3D printing in ER and trauma

3D printing, also known as additive manufacturing, has revolutionized the field of MSK radiology, particularly in emergency and trauma settings. With its ability to create detailed, patient-specific models from imaging data, 3D printing offers a range of benefits that enhance the diagnostic, planning, and treatment processes in MSK radiology. In emergency and trauma radiology, the speed and accuracy with which 3D printing can produce physical models of complex fractures, deformities, or surgical sites can significantly impact clinical decision-making and patient outcomes.^29^

3D printing in MSK radiology is primarily based on converting digital imaging data, such as CT or MRI scans, into three-dimensional physical models. These models provide a tangible representation of the anatomical structures involved, which can be used in various ways for both diagnosis and treatment. The process begins with the acquisition of high-resolution imaging data from CT, MRI, or even DECT. Advanced software then converts this data into a digital model, which is further processed to create a physical, three-dimensional object using materials like resin, plastic, or metal.

**Figure 3.**
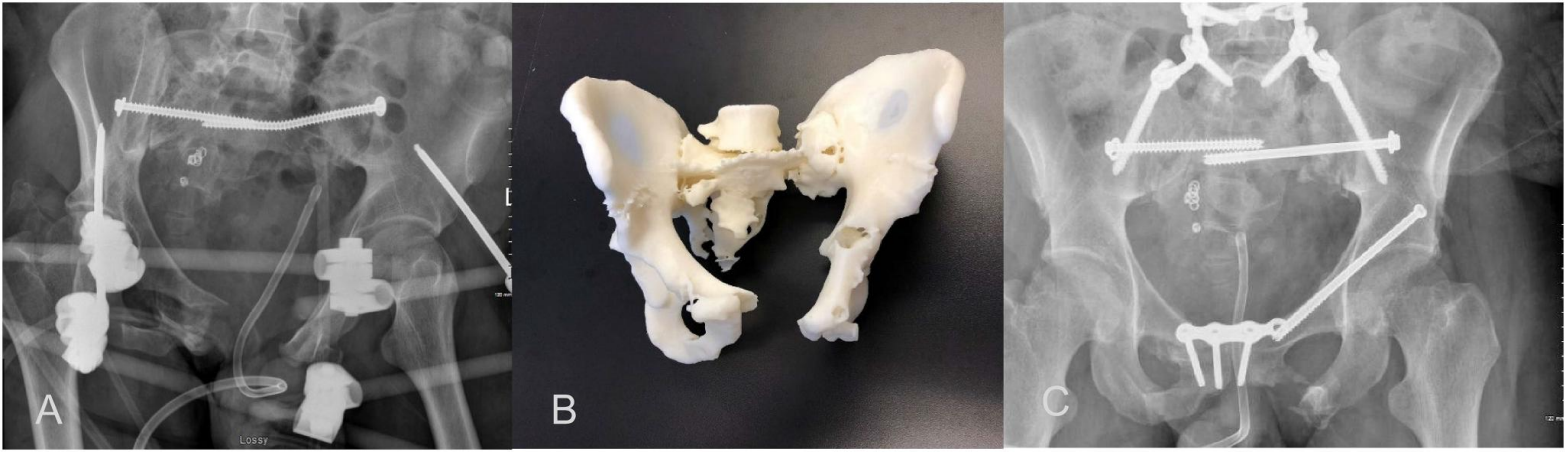
Patient presented with MVA with complex pelvic fracture (A). 3D printing for the pelvis was requested by the trauma team for surgical planning of hardware (B). A successful reconstruction surgery was performed—post operative radiograph (C).

The application of 3D printing in MSK radiology has gained significant traction in trauma and emergency settings, where rapid decision-making is critical for improving patient outcomes (Figure 3).

### Clinical applications of 3D printing in MSK radiology for trauma and emergency settings

1. *Preoperative Planning and Surgical Simulation:* One of the most impactful uses of 3D printing in trauma radiology is preoperative planning. The ability to generate accurate, patient-specific models of fractures, deformities, or anatomical irregularities enables surgeons and radiologists to plan more precisely. For example, a complex fracture in a trauma patient can be 3D printed, allowing the surgical team to evaluate the injury from different angles and better understand the fracture’s complexity. This also aids in determining the appropriate surgical approach, ensuring that critical structures are avoided and optimizing the placement of implants or fixation devices.^30–32^
  In cases of joint replacements or fractures involving multiple bone segments, the ability to visualize the entire anatomy of the affected area is invaluable. The physical model can be used to simulate surgical procedures, practice the surgery, and even test different treatment options to determine the best course of action before making any incisions.
2. *Guiding Implant Design and Placement:* For patients with complex fractures or deformities, 3D-printed models can be used to create customized implants or guides tailored to the individual’s anatomy. In emergency settings, where time is often of the essence, having access to a model that is directly correlated to the patient’s anatomical structures can lead to more precise implant placement and faster surgical intervention.^33^^,^^34^
  Custom implants designed from 3D models have proven particularly useful in patients with anatomical irregularities, previous surgeries, or nonhealing fractures. These custom-designed implants fit better, reduce the risk of complications, and improve overall outcomes in trauma surgery.^35^^,^^36^
3. *Educational Tool for Radiologists and Surgeons:* 3D-printed models serve as excellent educational tools for training radiologists, surgeons, and medical students in the nuances of complex MSK cases. By handling a physical model of a fracture or injury, medical professionals can gain a deeper understanding of anatomical variations, fractures, and surgical strategies.^37^
  In trauma radiology, where emergency decisions need to be made quickly, using 3D models to teach radiologists how to assess injuries accurately in a more hands-on manner can improve their diagnostic skills. In addition, these models can be used in simulations to train surgical teams, allowing them to practice in a realistic but controlled environment.
4. *Facilitating Communication Among Multidisciplinary Teams:* Effective communication between various healthcare professionals, including trauma surgeons, orthopedic specialists, radiologists, and anesthesiologists, is essential for optimal patient care in emergency and trauma situations. 3D-printed models offer a tangible way to convey complex imaging findings and treatment plans across teams. Rather than relying solely on two-dimensional images, 3D models allow for more effective interdisciplinary communication by providing all parties with a clear, shared understanding of the patient’s anatomy and injury.
  In complex trauma cases where multiple specialists are involved, using 3D models for consultations can improve the collaborative approach to patient care and streamline decision-making.
5. *Emergency Trauma and Fracture Evaluation:* In emergency trauma situations, where time is critical, 3D printing can play a role in quickly visualizing and assessing fractures, joint dislocations, and complex injuries. While traditional imaging techniques, such as CT and X-rays, provide valuable information, 3D printing allows radiologists and surgeons to assess the fracture or injury in three dimensions, which may help reveal important details that are not obvious on 2D images.
  For instance, fractures involving the pelvis, spine, or other areas with complex bone structures can be challenging to assess fully using conventional imaging. 3D-printed models provide more detailed anatomical representations, facilitating better understanding of the fracture’s alignment, severity, and potential complications.
6. *Personalized Orthotics and Prosthetics:* Another significant role of 3D printing in MSK radiology is the creation of personalized orthotics and prosthetics. Following traumatic injuries, patients may require custom-made splints, casts, or prosthetics that are designed to fit their specific anatomy. Using 3D imaging and printing, orthotic devices can be created with a high degree of precision, ensuring optimal fit and comfort for the patient.^38^
  In cases where a patient requires an immediate prosthetic after an amputation or trauma, 3D-printed models can expedite the process of designing and fabricating a customized prosthesis, reducing waiting times and enhancing patient satisfaction.
7. *Postoperative Monitoring and Assessment:* 3D printing can also be useful for postoperative assessment and monitoring, especially when evaluating the effectiveness of surgeries. Post-surgical 3D-printed models can help radiologists and surgeons assess the alignment of fractures, the placement of implants, and the healing process. By comparing preoperative and postoperative models, healthcare providers can identify complications early, such as implant failure, infection, or nonunion of fractures, allowing for more timely interventions.^39^
  In the case of joint replacements, for example, 3D-printed models can be used to track the long-term outcomes of implants, helping doctors determine whether revisions or adjustments are needed.

### Challenges and limitations of 3D printing in MSK radiology for trauma and emergency settings

1. *Cost and Accessibility:* One of the significant barriers to the widespread use of 3D printing in MSK radiology is cost. High-quality 3D printers and materials can be expensive, and not all medical centers have the resources to invest in this technology. Moreover, the expertise required to convert imaging data into accurate 3D models and interpret them effectively may require additional training.
2. *Time Considerations:* While 3D printing offers many advantages, it can be time-consuming, particularly in emergency trauma situations where quick decisions are required. Depending on the complexity of the fracture or injury, creating and printing the model may take valuable time that could be better spent on immediate surgical intervention.
3. *Technical Limitations of Models:* Despite the advances in 3D printing technology, certain technical limitations remain. For instance, the resolution of printed models may not always match the level of detail provided by imaging techniques like MRI or CT. While 3D-printed models are highly useful, they may not fully replicate the tissue density or subtle variations in anatomy seen on imaging scans.

### The role of MRI in acute trauma imaging

**Figure 4.**
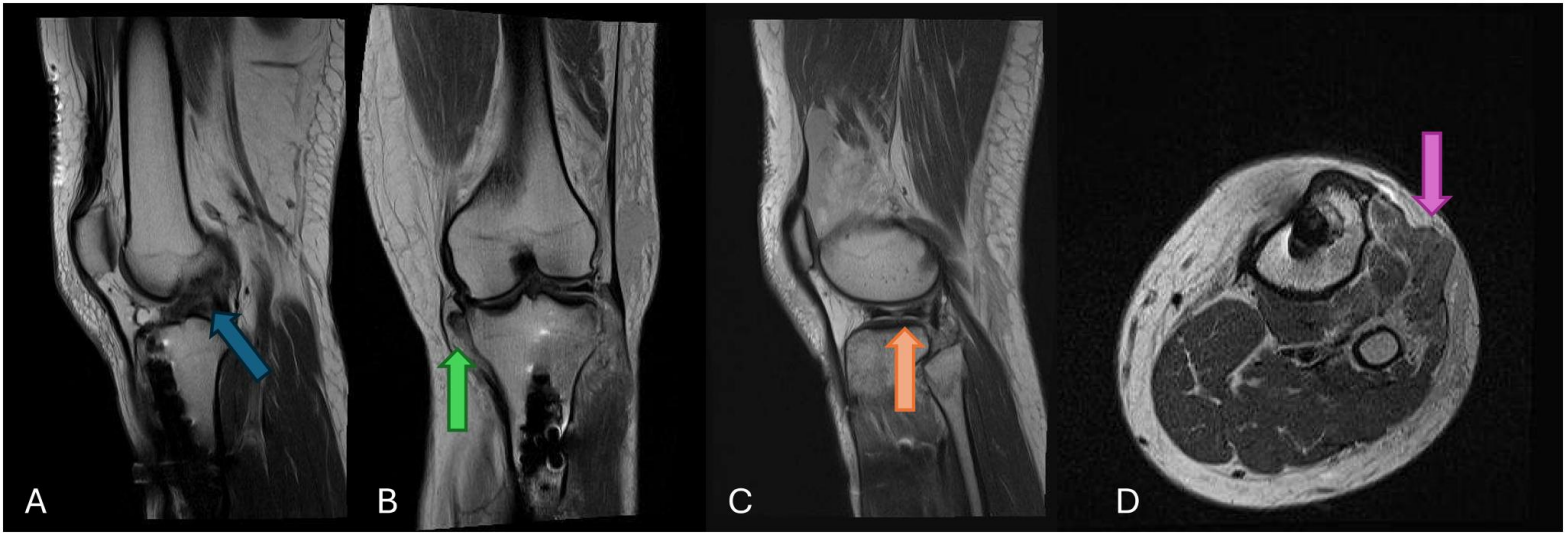
Patient presented in ER with poly trauma after motor vehicle accident. The baseline lower limb part of the whole body CT was limited due to artifacts. Due to high level of suspicion for soft tissue injuries MRI was performed which detected high grade partial thickness ACL tear (image A), complete tear of the distal MCL with retraction (image B), lateral meniscal tear (image C) and fascial tear of the anterior compartment of the proximal leg with herniation of the extensor digitorum longus and tibialis anterior muscles (image D).

**Figure 5.**
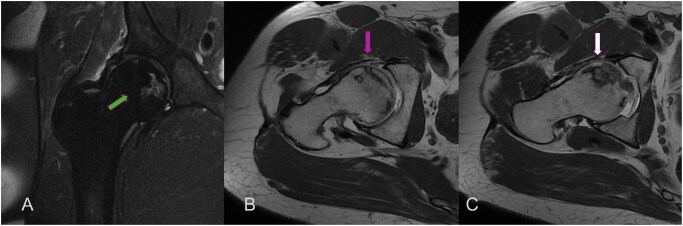
Patient presented with hip pain. MR Arthrogram was performed for the clinical question of labral tear. MRI conformed the anterior labral tear. Incidental AVN of the femoral head was also seen.

Magnetic resonance imaging (MRI) is an indispensable tool in MSK radiology, particularly in the trauma setting. With its exceptional ability to visualize soft tissues, including muscles, tendons, ligaments, cartilage, and bone marrow, MRI offers unique advantages over traditional imaging modalities like X-rays and CT scans. In trauma imaging, MRI plays a crucial role in diagnosing soft tissue injuries, evaluating bone marrow changes, detecting occult fractures, and providing detailed information for surgical planning and rehabilitation^40^ (Figures 4 and 5).

MRI excels in trauma imaging because of its high spatial resolution and superior contrast between soft tissues and bone. Unlike X-rays and CT scans, which primarily focus on bone structures, MRI provides detailed images of both hard and soft tissues, making it invaluable in assessing complex trauma, especially when soft tissue involvement is suspected.^41^^,^^42^

MRI is particularly effective for evaluating:

- *Ligamentous injuries*: MRI is the gold standard for assessing ligament tears and sprains, particularly in joints such as the knee, shoulder, and ankle.
- *Tendon injuries*: It provides high-resolution images of tendon tears, strains, and degeneration, which are common in traumatic injuries.
- *BME*: MRI is the most sensitive imaging modality for detecting BME, which often indicates trauma-related bone injuries, stress fractures, or inflammation.
- *Occult fractures*: MRI can identify fractures that may not be visible on standard radiographs or even CT, especially when the fracture is subtle or in areas with complex anatomy (eg, scaphoid fractures in the wrist).
- *Soft tissue hematomas*: MRI is also critical in identifying and evaluating the extent of hematomas, especially in cases involving muscle and tendon injuries.

### Clinical applications of MRI in acute trauma settings

1. *Evaluation of BME:* One of the most important applications of MRI in trauma imaging is the detection of BME. BME is a non-specific finding that often occurs after trauma and is associated with various MSK pathologies, including fractures, osteoarthritic changes, and infections. MRI’s ability to detect and quantify BME provides valuable diagnostic information, helping clinicians assess the severity and prognosis of traumatic injuries.^43^
  BME is best seen on fat-saturated T2-weighted MRI sequences, which demonstrate areas of increased signal intensity, suggesting fluid accumulation in the bone marrow due to injury or inflammation. In the trauma setting, the presence of BME can indicate a stress fracture, bone contusion, or early-stage fracture healing.
2. *Soft Tissue Injuries:* MRI is the imaging modality of choice for diagnosing ligamentous and tendon injuries, which are common in trauma cases. Ligaments, tendons, and cartilage, being primarily composed of soft tissue, are poorly visualized on X-rays and CT scans. MRI’s high contrast resolution between soft tissues allows for a detailed evaluation of ligament and tendon tears, sprains, or avulsions. MRI is also highly effective in detecting articular cartilage injuries, which can occur in joint dislocations or high-impact trauma. Cartilage injuries can lead to joint instability and long-term complications, including osteoarthritis, making early diagnosis crucial.^44^^,^^45^
3. *Evaluation of Occult Fractures* One of the key roles of MRI in trauma radiology is the detection of occult fractures. These are fractures that are not visible on initial X-rays but can cause significant pain and functional impairment. MRI is far more sensitive than X-ray for identifying subtle fractures, particularly in areas like the scaphoid, vertebral body, and pelvis.^46^^,^^47^
  Similarly, MRI can be used to evaluate stress fractures, which might not be detected on standard CT or X-ray in their early stages. In trauma patients who present with persistent pain and negative initial imaging, MRI can help confirm a diagnosis and guide treatment.
4. *Post-Traumatic Osteomyelitis and Infection* In trauma patients, infection is a potential complication, especially in cases of open fractures, surgical wounds, or compromised immune systems. MRI is an essential tool in detecting osteomyelitis, a bone infection often caused by trauma-related bacteria. MRI’s ability to evaluate both soft tissue and bone makes it ideal for identifying soft tissue abscesses, bone marrow infections, and bone destruction.
  In cases of osteomyelitis, MRI can reveal areas of abnormal bone marrow signal (BME), cortical irregularities, and surrounding soft tissue infection. The use of gadolinium contrast in MRI can also improve the sensitivity and specificity of detecting infection.^48^
5. *Assessment of Muscle Injuries:* Muscle injuries, including strains and contusions, are common in trauma patients, especially in those involved in sports or high-impact accidents. MRI is effective at detecting muscle tears, hematomas, and muscle fiber disruption. Unlike standard CT and X-ray, MRI provides excellent soft tissue contrast, enabling differentiation between different types of muscle injuries.
  MRI can help classify muscle injuries by severity, identify associated hematomas, and guide the timing for rehabilitation. The role of MRI in muscle injuries is especially important in high-performance athletes or individuals who require quick recovery and precise treatment planning.^49–51^
6. *Joint Dislocations and Subluxations:* MRI is often used to evaluate the soft tissue damage associated with joint dislocations or subluxations, where ligaments, tendons, and cartilage may be disrupted allowing for optimal treatment planning, whether surgical or conservative.^52^
7. *Preoperative Planning*: For complex trauma cases requiring surgery, MRI provides valuable preoperative information, particularly for surgical planning in cases involving soft tissue injury. MRI allows surgeons to assess the exact location, size, and involvement of soft tissue injuries, facilitating more accurate decision-making regarding surgical approaches.
8. *Evaluation of Non-traumatic Injuries:* MRI plays a crucial role in diagnosing acute non-traumatic conditions such as spinal cord compression, disc herniation, osteomyelitis, and septic arthritis. MSK radiologists interpret marrow signal changes, soft tissue extension, and abscess formation, often making MRI the gold standard in infection and inflammatory emergencies.^53^

### Limitations of MRI in trauma imaging

- *Limited availability*: MRI is not always available in all trauma settings, particularly in remote or emergency locations. Furthermore, MRI machines are often less portable than CT scanners or X-ray machines, which may delay imaging for critically ill or unstable patients.
- *Time-consuming*: MRI exams can take longer to complete than other imaging modalities, which can be a limitation in acute trauma settings where rapid decision-making is necessary.
- *Contraindications*: MRI is contraindicated in patients with certain implants (e.g., pacemakers, metal fragments) or those who are claustrophobic, which may limit its use in some trauma patients. Particularly in the context of trauma, patients with suspected or confirmed metallic foreign bodies due to the strong magnetic field, which can cause displacement, rotation, or heating of ferromagnetic objects, leading to serious injury. This risk is particularly critical with intraorbital metallic fragments, vascular clips, or shrapnel, where migration can damage vital structures.^54^

## The role of portable MRI in emergency and trauma settings

Portable MRI is an emerging technology although major role is in neuroimaging but also helps in MSK imaging.^5^^,^^55^

### Advantages of portable MRI in acute trauma care

1. *Assessment of Spinal Cord and Soft Tissue Injuries*
  - Spinal cord injuries (SCI) require early and precise diagnosis to determine the need for surgical intervention.
  - Portable MRI provides high-resolution imaging of spinal cord edema, ligamentous injuries, and epidural hematomas, improving decision-making for trauma surgeons.
2. *Alternative to Ionizing Radiation in Repeated Imaging*
  - Trauma patients often undergo multiple imaging studies, leading to significant cumulative radiation exposure.
  - Portable MRI, being radiation-free, serves as a safer alternative for follow-up imaging in pediatric patients, pregnant patients, and individuals requiring multiple scans.
3. *Detection of BME in Occult Fractures*
  - Conventional radiographs may miss occult fractures, particularly in the hip, pelvis, and extremities.
  - Portable MRI can detect BME, a key indicator of microfractures, stress fractures, and early osteonecrosis, providing a bedside alternative to standard MRI in cases where DECT is unavailable.
4. *Soft Tissue and Joint Injury Evaluation*
  - Portable MRI enhances the evaluation of ligamentous and meniscal injuries in acute knee and shoulder trauma.
  - Point-of-care imaging in sports medicine and emergency settings helps determine the need for urgent intervention versus conservative management.
5. *Faster Imaging Workflow in Mass Casualty Events*
  - In disaster scenarios or military field hospitals, where conventional imaging infrastructure is limited, portable MRI provides rapid assessment of trauma patients, improving triage efficiency and guiding treatment decisions.

### Limitations

While portable MRI offers significant advantages, current low-field systems have limitations in spatial resolution and imaging speed compared to conventional high-field MRI (1.5T or 3T). However, ongoing advancements in machine learning-based image reconstruction and hardware miniaturization are expected to enhance image quality, making portable MRI an increasingly valuable tool in trauma care.

## The role of PCCT

Photon-counting computed tomography is an emerging imaging technology that represents a significant advancement over conventional CT. Unlike traditional CT, which converts X-ray photons into visible light before detecting them, PCCT directly counts individual X-ray photons and measures their energy levels. This results in improved spatial resolution, reduced noise, better tissue contrast, and lower radiation doses.^56^^,^^57^

For MSK radiology, PCCT has the potential to revolutionize imaging in trauma and emergency settings by enhancing fracture detection, reducing artifacts, and improving soft tissue visualization.^58–60^

### Advantages of PCCT in MSK trauma imaging

1. *Superior Fracture Detection and Bone Microstructure Analysis:* PCCT provides ultra-high spatial resolution imaging, allowing for detailed visualization of trabecular bone microarchitecture. This capability is particularly beneficial for detecting occult fractures that may be missed on standard CT, such as scaphoid and metatarsal fractures in wrist and foot injuries, subtle tibial plateau fractures in knee trauma, and insufficiency fractures in elderly and osteoporotic patients. The enhanced cortical bone delineation enables better assessment of fracture displacement, comminution, and healing.^59–61^
2. *Improved Bone Marrow Imaging:* PCCT enables material decomposition, which enhances the differentiation of bone marrow components. This feature allows for early detection of BME, making PCCT a potential alternative to MRI in trauma patients. It aids in identifying conditions such as acute vertebral compression fractures vs. chronic fractures, early stress injuries in athletes and osteonecrosis and bone infarcts in sickle cell disease or corticosteroid use.
3. *Reduced Metal Artifacts in Post-Surgical and Trauma Patients:* Trauma patients often require orthopedic hardware, prostheses, or surgical plates that create beam-hardening artifacts on standard CT.^62^
4. *Enhanced Soft Tissue Imaging and Tendon/Ligament Assessment:* While MRI remains superior for soft tissue evaluation, PCCT’s ability to differentiate materials at different energy levels provides improved contrast for tendon, ligament, and cartilage injuries.^63^
5. *Lower Radiation Dose: Safer Imaging for Trauma Patients:* One of the key advantages of PCCT is its ability to achieve superior image quality at a lower radiation dose compared to conventional CT. This is particularly beneficial in Pediatric trauma imaging, where radiation exposure is a major concern, polytrauma patients requiring multiple scans during their hospital stay and follow-up imaging, allowing repeated scans with minimal radiation risk.^58^^,^^60^

### Future applications and clinical impact

- *AI Integration:* The integration of PCCT with AI-driven analysis could streamline trauma imaging, reducing interpretation time and enhancing diagnostic accuracy.
- *Dynamic Imaging:* Future PCCT advancements may enable dynamic musculoskeletal imaging, allowing real-time assessment of joint function and biomechanics in trauma patients.
- *Emergency Room Workflow Enhancement:* As PCCT technology becomes more accessible, its speed and diagnostic accuracy could streamline ER workflows.

## The role of MSK radiologist in pediatric trauma and emergency

In the pediatric emergency setting, evaluating both traumatic and non-traumatic conditions require rapid and accurate imaging further highlighting the role of MSK radiologist. Traumatic injuries such as fractures, growth plate disruptions, and soft tissue injuries are common in children and demand careful imaging evaluation to avoid misdiagnosis and long-term functional impairment.^64^ While radiographs remain the first-line modality, CT and DECT are reserved for complex fractures and polytrauma.^2^ MRI is indispensable for diagnosing occult fractures, ligamentous injuries, infections, and inflammatory conditions, as it provides excellent soft tissue detail without radiation exposure.

Ultrasound serves as a valuable tool for diagnosing fractures in pediatric patients, offering a radiation-free alternative, particularly in the evaluation of long bone fractures, such as those involving the forearm, clavicle, and femur, where it can reliably detect cortical disruptions, step-offs, and periosteal hematomas.^65^^,^^66^ Ultrasound is especially advantageous in children due to their thinner soft tissues and lower bone mineralization, which allow for better visualization of fracture lines compared to adults.^67^ In addition to initial diagnosis, ultrasound can also be used to monitor healing and detect complications such as malunion or infection without repeated radiation exposure.^68^

Collectively, these modalities enable MSK radiologists to differentiate acute trauma from mimics such as infection, inflammatory arthropathies, or neoplasms in the pediatric ER.

## Conclusion

MSK radiologists are essential in the ER and trauma settings ensuring rapid and accurate diagnoses that guide clinical management. While DECT has expanded the role of CT in detecting BME, infection, and soft tissue injuries, MRI remains the gold standard for detailed soft tissue and bone marrow evaluation. The future of trauma imaging is poised for further innovation with the advent of PCCT, which promises to improve diagnostic accuracy while reducing radiation exposure. As technology continues to evolve, MSK radiologists will play an increasingly pivotal role in integrating these advanced imaging modalities to optimize patient care in emergency and trauma settings.
